# Assessment of Resistance Mechanisms and Clinical Implications in Patients with KRAS Mutated-Metastatic Breast Cancer and Resistance to CDK4/6 Inhibitors

**DOI:** 10.3390/cancers13081928

**Published:** 2021-04-16

**Authors:** Lucrezia Raimondi, Filippo Maria Raimondi, Marta Pietranera, Arianna Di Rocco, Laura Di Benedetto, Evelina Miele, Rachele Lazzeroni, Giuseppe Cimino, Gian Paolo Spinelli

**Affiliations:** 1 U.O.C. Territorial Oncology of Aprilia, Sapienza University of Rome, 04011 Aprilia, Italy; lucrezia.raimondi@uniroma1.it (L.R.); cimino@bce.uniroma1.it (G.C.); 2 E-Campus University, 00100 Rome, Italy; filippo3006@gmail.com; 3 Centro Medico Diagnostico Salus, Via Cadorna 8, 00053 Civitavecchia, Italy; dottpietranera.m@gmail.com; 4 Department of Public Health and Infectious Diseases, Sapienza University of Rome, 00100 Rome, Italy; Arianna.dirocco@uniroma1.it; 5 BIOS SpA, Via Domenico Chelini 39, 00197 Roma, Italy; lauradibenedetto.ldb@gmail.com; 6 Department of Paediatric Haematology/Oncology Cell and Gene Therapy, Bambino Gesù Children’s Hospital, IRCCS, 00165 Rome, Italy; evelina.miele@opbg.net; 7 Department of Medical and Surgical Scieces and Translational Medicine, “Sapienza” University of Rome, 00100 Rome, Italy; r.lazzeroni@hotmail.it

**Keywords:** metastatic breast cancer, targeted therapy, KRAS, CDK4/6 inhibitors, liquid biopsy, ddPCR, resistance mechanisms

## Abstract

**Simple Summary:**

Palbociclib in combination with fulvestrant is used globally to treat metastatic breast cancer, but it was recognized that not all patients benefit from this combination of drugs. However, the predictive factors remain unknown. Here, we show KRAS ctDNA levels as predictive mechanisms of resistance to palbociclib and fulvestrant, and their association with the time to treatment discontinuation of the above treatment. These observations shed light on the potential clinical applications of ctDNA analysis in this setting of patients, in order to provide critical information about tumour dynamics, and to predict who will take advantage from CDK4/6 inhibitors.

**Abstract:**

Despite therapeutic improvements, resistance to palbociclib is a growing clinical challenge which is poorly understood. This study was conducted in order to understand the molecular mechanisms of resistance to palbociclib, and to identify biomarkers to predict who will take advantage from cyclin-dependent kinase 4/6 inhibitors (CDK4/6i). A total of about a thousand blood samples were collected from 106 patients with hormone receptor positive (HR+) human epidermal growth factor receptor 2 (HER2) negative metastatic breast cancer who received palbociclib in combination with fulvestrant as the first-line metastatic therapy enrolled in this study. The genotyping of their plasma cell-free DNA was studied, including serial plasma samples. Collectively, our findings identify the appearance of KRAS mutations leading to palbociclib resistance acquisition within 6 months, and provide critical information for the prediction of therapeutic responses in metastatic breast cancer. By monitoring KRAS status through liquid biopsy, we could predict who will take advantage from the combination of palbociclib and fulvestrant, offering highly-individualized treatment plans, thus ensuring the best patient quality of life.

## 1. Introduction

In the last few years, therapeutic strategies for metastatic breast cancer (mBC) have deeply changed. The introduction of cyclin-dependent kinase 4 and 6 inhibitors (CDK 4/6i) in combination with endocrine therapy for the treatment of women with hormone receptor-positive (HR)/human epidermal growth factor receptor 2-negative (HER2) breast cancer has radically changed the treatment of patients with breast cancer, improving their health-related quality of life and life expectancy, with a high response rate and longer median progression-free survival (PFS) and overall survival (OS) [1,2,3,4,5,6,7,8].

Therefore, all of the current guidelines recommend the consideration of a CDK4/6 inhibitor-based first line therapy for mBC.

Albeit that these are promising results with indubitable clinical benefits, some open issues remain to be clarified for the full exploitation of the potential of these drugs: a substantial number of patients are intrinsically refractory to CDK4/6i and do not respond at all or only minimally; other ones, who initially respond, escape from this therapy and experience a survival benefit in the range of a few months. Moreover, acquired resistance has become a clinical problem. The molecular profiling of patients’ tumors can direct treatment decisions by alerting us to the emergence of treatment resistance and disease relapse, but no specific and predictive biomarkers are currently available for the accurate detection of resistance to CDK4/6i.

Blood-based liquid biopsy, as an alternative to tumor biopsy, might improve the management of patients with mBC, as numerous studies have demonstrated its usefulness for cancer prognosis, diagnosis, and interestingly for the prediction of response or resistance to administered therapeutics [9,10,11,12].

Several studies have suggested that a mutant KRAS protein may induce cyclin D1 overexpression through the constitutive activation of the RAS-MEK-ERK pathway, resulting in cell growth and cancer development [13]. Luangdilok et al. (in 2019) showed that a downstream effector of KRAS is Cyclin D1/CCND1, which seems to control cell division by regulating CDK4/6 activity during the G1-S transition of the cell cycle [14,15]. Nevertheless, no previous studies have been conducted to evaluate the potential predictive role of KRAS status on the emergence of resistance to CDK4/6i. Herein, for the first time, we present the results of our study to investigate the predictive and prognostic role of the KRAS status in patients with metastatic breast cancer who were in treatment with a CDK4/6 inhibitor, palbociclib and fulvestrant, with the aim of understanding the clinical and molecular indicators that might suggest a specific molecular mechanism of resistance and a treatment approach.

## 2. Materials and Methods

### 2.1. Study Population

Patients with histologically confirmed HR-positive/HER2-negative mBC who had disease progression after previous endocrine therapy, treated with palbociclib in combination with fulvestrant, were enrolled in the study between December 2017 and March 2020.

Patients were eligible for the study if they had biopsy-proven metastatic breast cancer with tissue available for standard genotyping (or a planned repeat biopsy), and were naïve to treatmentwith CDK4/6 inhibitors. A computed tomography-guided bone biopsy, a liver biopsy, or a lymph node biopsy was performed. Patients with insufficient clinical data, or who discontinued treatment after the first cycle of CDK4/6 inhibitors and fulvestrant, were excluded from the study. The study was performed in accordance with the International Conference on Harmonisation Guidelines on Good Clinical Practice, and with the Declaration of Helsinki. Despite the fact that this study collected retrospective clinical data with no risk to the participants, written informed consent was obtained for the collection of plasma and the profiling of the tumour DNA before any study-related procedures occurred. All of the patients underwent blood collection after their study enrollment before starting palbociclib and fulvestrant, and then underwent follow-up blood draws every two months during the course of treatment (range of longitudinal samples collected: 2–10).

The purpose of the study was to determine if *KRAS*-mutated ctDNA detection in patients diagnosed with metastatic breast cancer could be used as a predictive factor for resistance to CDK4/6i. Progression Free Survival (PFS) was defined as the time from enrollment to disease progression according to RECIST 1.1 or death from any cause.

### 2.2. Molecular Analyses

When this study was conducted, all of the analyses and data on the tumour KRAS status for all of the assessable patients were already available. DNA was extracted from formalin-fixed, paraffin-embedded (FFPE) tissue sections from resection samples, and the data were analyzed using standard PCR-based techniques. All of the mutations detected were confirmed on PCR and sequencing analysis. A droplet digital polymerase chain reaction (ddPCR) was performed on the same DNA which was previously extracted for standard PCR-based techniques, in order to screen for the KRAS mutations found in the plasma but not previously detected in the tissue. The plasma samples were collected a few weeks after the biopsy of the metastatic lesion.

The plasma was collected with the patients’ consent: all patients underwent an initial paired blood collection after study enrolment. These two tubes of blood underwent plasma isolation, and the extraction of circulating cell-free DNA and ddPCR-based genotyping. The first tube of blood was processed and analyzed immediately; the second one underwent planned variations designed to simulate real-world testing conditions.

According to the manufacturer’s protocol, circulating cell-free DNA was extracted from 3 mL plasma using a QIAmp Circulating Nucleic Acid Kit (QIagen, Hilden, Germany). The KRAS status in the plasma was analyzed using the Bio-Rad QX200 ddPCR system (BioRad^®^, Hercules, CA, USA) using a commercially-available Prime PCR KRAS kit for ddPCR KRAS Screening Multiplex. With Quanta-Soft software, the number of positive and negative droplets (number of copies/mL) was measured; the target number of copies/mLwas calculated using a Poisson distribution. For data reproducibility, the analysis of KRAS status in the plasma was performed in triplicate in a short time interval. The laboratory personnel performing the plasma ddPCR were blinded to the tissue genotyping results.

The fractional abundance—which is the percentage of mutant KRAS—was calculated as the ratio of drops positive for the mutant allele to drops positive for the wild-type allele plus drops positive for the mutant allele.The sensitivity cut-off for the ctDNA detection assay was set at the lower limit of 0.02% mutant alleles. The plasma genotyping results were comparedwith the tissue genotyping results from the biopsy.

### 2.3. Statistical Analysis

The continuous variables are presented as mean ±SD or median ± interquartile range (IQR), depending on the shape of the distribution curve. The categorical variables were summarized with counts and percentages, and were compared by Χ2 or Fisher’s exact tests. The PFS was estimated through Kaplan–Meier estimates, and the effects of the predictors were assessed through log-rank tests and univariate Cox regression. The following variables were analyzed in patients who underwent palbociclib and fulvestrant treatments: age (≤65 years versus >65 years); histology (ductal versus lobular); pathological differentiation (well versus moderate and others); number metastatic sites (one versus two or more); lymph node metastasis (negative versus positive); liver metastasis (negative versus positive); bone metastasis (negative versus positive); the presence of KRAS-mutated ctDNA before starting treatment (negative versus positive); and the emergence of KRAS-mutated ctDNA upon longitudinal monitoring.

A multivariable Cox regression model was estimated, in which the final set of predictors was selected based on the minimization of the Akaike Information Criterion in the forward selection stages. The significance was fixed at the 0.05 level. All of the analyses were performed using R version 3.5.1 (URL https://www.R-project.org) (accessed on 31 July 2018).

## 3. Results

### 3.1. Baseline Characteristics

A total of 264 patients, all of them Caucasian, with HR-positive/HER2-negative mBC with sensitivity to previous endocrine therapy were enrolled in the study. Sensitivity to previous endocrine therapy was defined as the receipt of at least 24 months of adjuvant endocrine therapy before recurrence. The patients were treated with the standard-of-care combination palbociclib plus fulvestrant as first-line metastatic therapy, and before starting treatment, they were assessable for the analysis of KRAS mutations in tumour tissue and in their ctDNA. The patients (*n* = 69) who did not complete their tissue genotyping or initial blood sampling (*n* = 25) were excluded from the analysis. An additional 59 patients did not have sufficient tissue available for *KRAS* testing, and were excluded. An additional five patients withdrew consent after enrollment. Of the 106 eligible patients, the baseline demographics and disease characteristics are summarized in Table 1.

KRAS status evaluation was previously performed on the tumour tissue, then in plasma; the information on the tissue KRAS status was available for the totality of the ctDNA-assessable patients (*n* = 106). Forty patients (37.7%) had a detectable KRAS mutation (^mut^KRAS) in codon G12V, G12D or G13D, and four patients (3.7%) had less common ^mut^KRAS on tumour tissue (i.e., G12A = 2; A146T = 1; G12R = 1). In two patients, two mutations were found (i.e., G12D/ G13D and G12V/ G12A). Fifty-two patients (49%) had KRAS wild-type. At the baseline, 53.7% of the patients were carriers of at least one ^mut^KRAS in ctDNA: 57 subjects were carriers of p.G12V, p.G12D or p.G13D; four patients had less common KRAS mutations, and two patients had two mutations (the same ones found in the tissue).

**Figure 1:**
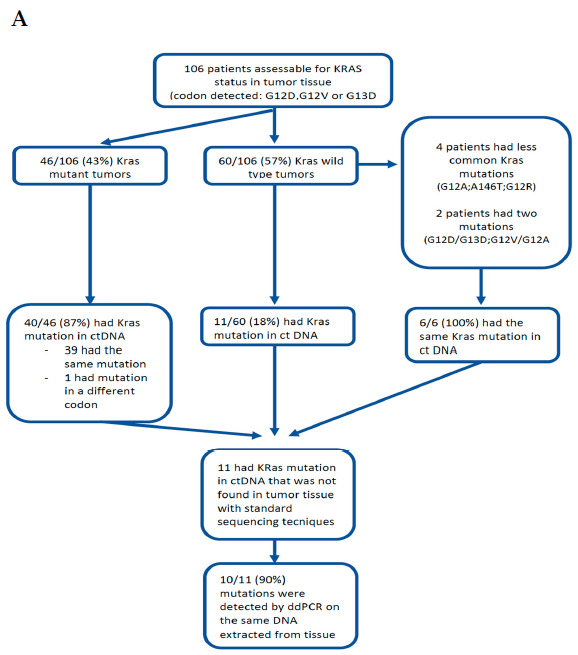

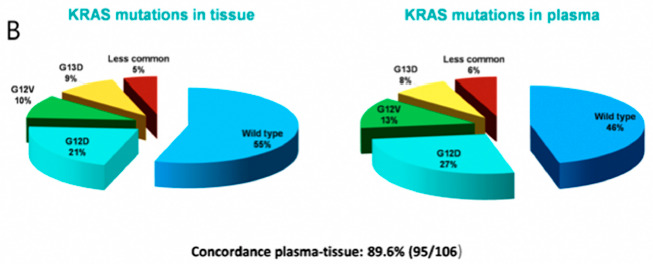
In Figure A, we present the study flow diagram and tissue availability in the ctDNA assessable population. In Figure B, we present the frequency of the most frequently-mutated *KRAS* mutation in tissue (**A**) and plasma (**B**) of patients who were assessable for the analysis of ctDNA. Concordance plasma-tissue was 89.6%.

Forty-nine patients were negative for ctDNA^mut^KRAS at baseline; six of them turned to positivity several months after the treatment’s start (Figure 1). In 11 patients, the ctDNA analysis revealed ^mut^KRAS not previously found in the tumour tissue. Of note, using ddPCR to analyse the same DNA previously extracted from tissue and assessed by standard PCR-based techniques, in 10/11 samples (90.9%) ^mut^KRAS were found. In the remaining patient, with no ^mut^KRAS identified in their tissue, the allele frequency in their plasma was 0.07. The concordance between the tissue analysis and liquid biopsy was 89.6% (95/106).

### 3.2. Monitoring ^mut^KRAS ctDNA during Treatment and Correlation with the Outcome and Resistance to CDK4/6 Inhibitors

Objective responses were defined following the RECIST criteria v.1.1, and the drug response was assessed every two months until the progression of the disease.

Six months after starting treatment, the objective response rate (ORR) was 18%, and the clinical benefit rate (CBR) was 46%. Nineteen (18%) patients achieved a partial response (PR), 29 (27%) achieved a stable disease (SD), and 58 (55%) had a progression of the disease (PD). The patients with PR and SD had KRAS wild-type (median value, 0 copies/mL), in contrast to the patients with PD in whom KRAS was mutated (median value, 80 copies/mL) (*p* < 0.0001). Only one patient with KRAS wild type ctDNA had PD. Six months after starting the treatment, in the KRAS wild-type ctDNA cohort, the ORR was 38.8% and the CBR was 97%; these results are consistent with ones reported in the PALOMA-2 and PALOMA-3 trials. The median duration of response (DOR) was 3 months for the ^mut^KRAS ctDNA group, and was not achieved in KRAS wild-type group (*p* < 0.0001).

Eighteen months after starting treatment, according to the radiological evaluation, all of the patients displaying ^mut^KRAS ctDNA (57 baseline ^mut^KRAS, one baseline KRAS wild-type, and eight wild-type that became ^mut^KRAS) had disease progression.

**Figure 2:**
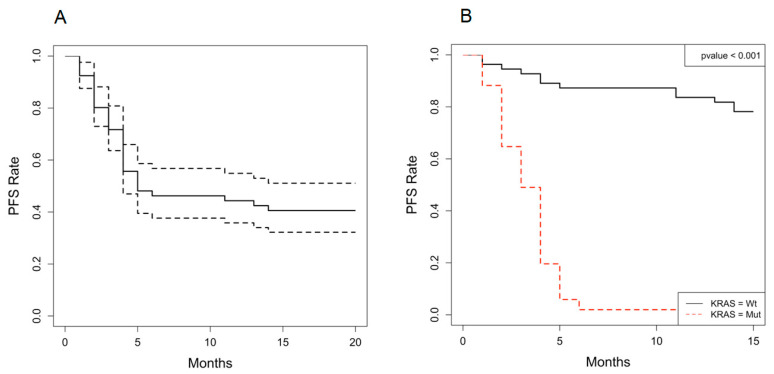
(**A**) At the 18-month follow up [1-NA], (**B**) the patients with *KRAS*-mutated ctDNA had a median PFS of 3 months (1–6 months, 95%CI 0.8–3.6), contrary to those with no detection of *KRAS* ctDNA whose PFS had not yet been reached (*p <* 0.001).

A statistically significant difference in PFS between patients displaying ^mut^KRAS ctDNA vs KRAS wild-type was observed. The median PFS of patients treated with palbociclib and fulvestrant was significantly better in the KRAS wild-type ctDNA group (*n* = 49; median, 17.8; range: 17.2—not achieved) than in the ^mut^KRAS group (*n* = 57; median PFS, 3 months, range 1–6.1 months, 95%CI 0.8–3.6) (HR, 20.746;95%CI,9.576–44.946; *p* < 0.001). Figure 2 depicts the PFS curves in the whole-study population.

Interestingly, the changes in the KRAS status provided critical information for the prediction of the therapeutic responses. The evidence of ^mut^KRAS ctDNA in the longitudinal tests was significantly associated with worse PFS, recurrence within 6 months (Fisher’s exact test, *p* < 0.0001), and resistance to palbociclib and fulvestrant (*p* = 0.001). ^mut^KRAS ctDNA could be used both as a predictive and prognostic biomarker.

### 3.3. Tumour Mutation Burden Is Significantly Associated with Increased Circulating ^mut^KRAS ctDNA Number of Copies/mL

**Figure 3:**
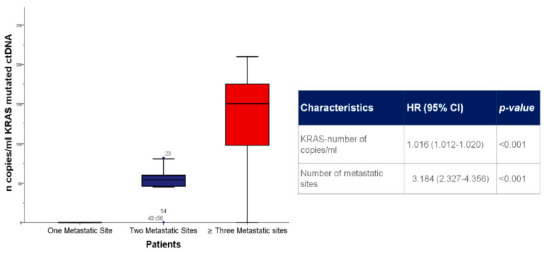
Tumour mutation burden was significantly associated with an increased circulating number of copies/mL of *KRAS*-mutated ctDNA.

Correlating the circulating ^mut^KRAS ctDNA number of copies/mL with the tumoral burden, higher ^mut^KRAS copies/mL at baseline were significantly associated with a higher number of metastatic sites. Patients with only one metastatic site had KRAS wild-type (median of 0 copies/mL); the median number of copies/mL for the two- or three-sites metastases positive subgroups were 50.1 (range, 24–56) and 127.8 (range, 91–169) copies/mL, respectively, with a statistically significant difference between the two groups (HR, 3.184; 95%CI 2.327–4.356; *p* > 0.001). A statistically significant association was observed between KRAS status and the site of metastasis. A higher level of lymph node involvement and liver metastases have been observed in ^mut^KRAS ctDNA patients (*p* < 0.001), as illustrated in Figure 3. No significant association was found comparing the KRAS status with bone metastases (*p* = 0.745).

### 3.4. Univariable and Multivariable Analyses of PFS among Patients Treated with Palbociclib and Fulvestrant

**Figure 4:**
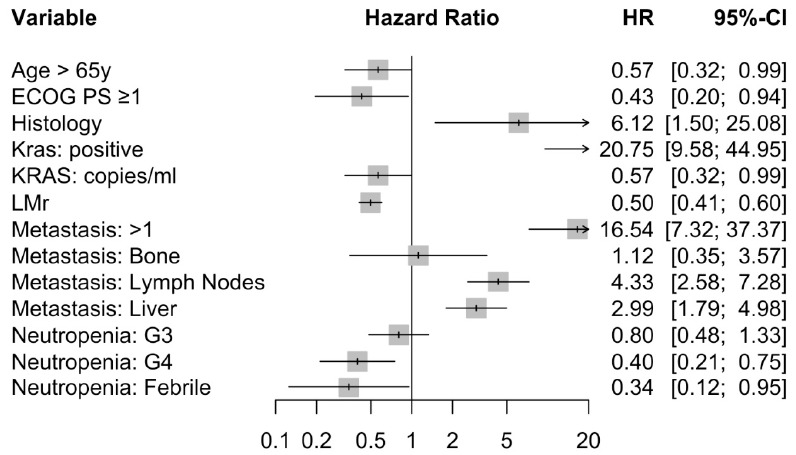
Forest-plot.

Table 2 presents independent demographic and clinical–pathological variables. The univariable analysis of each clinical and biological factor of PFS showed that the LMr (HR, 0.497, 95% CI, 0.411–0.601; *p* < 0.001), ^mut^KRAS ctDNA (HR, 20.743, 95% CI, 9.576–44.946; *p* < 0.001),KRAS number of copies/mL(HR, 1.016, 95% CI, 1.012–1.020; *p* < 0.001), number of metastatic sites (HR, 3.184, 95% CI, 2.327–4.356; *p* < 0.001), and presence of lymph node and liver metastases (HR, 4.334, 95% CI, 2.580–7.280; *p* < 0.001 and HR, 2.987, 95% CI, 1.792–4.980; *p* < 0.001, respectively) were significantly associated with PFS. The multivariable analysis of these factors showed that KRAS-wild type ctDNA (HR, 2.831, 95% CI, 1.060–7.558; *p* = 0.038) and Eastern Cooperative Oncology Group Performance Status (ECOG PS) (HR, 0.429, 95% CI, 0.195–0.942; *p* = 0.035) were significantly associated with a favorable PFS (Figure 4).

## 4. Discussion

In the last few years, while the potential applications of ctDNA in colon and prostate cancers have been subjects of a steadily-increasing number of investigations, the clinical usefulness of liquid biopsy in the mBC setting has not, so far, been intensively investigated, and much less in the era of CDK4/6i.

In modern oncology, screening for tumour molecular abnormalities is becoming increasingly important for the purposes of guiding clinicians in the decision-making process.

Notwithstanding the above, clonal evolution and intra-tumour heterogeneity have been recognized to represent tissue-based tests’ limits: genetic tests conducted on a small amount of tumour represent a suboptimal portrait of the tumour’s molecular profile [16]. In addition, single-lesion biopsies may not capture heterogeneity of resistance, missing alterations that might drive treatment failure, especially in plurimetastatic patients. In order to overcome the aforementioned limitations, liquid biopsy has emerged as an increasingly valid alternative analytic method which is able to provide a real-time exhaustive characterization of the cancer genome, offering the ability to dynamically monitor the emergence of resistance mechanisms in real-time, and to adjust therapy accordingly [17,18,19].

Despite the fact that the PALOMA-3 trial showed longer overall survival (34.9 months vs. 28.0 months) in women with mBC treated with palbociclib in combination with fulvestrant than fulvestrant alone, sooner or later the majority of the patients acquire resistance to CDK4/6i in the course of treatment [5]. Overcoming resistance and probing efficacy predictors to select the patients who should obtain the most benefit from these drugs are the major challenges for clinicians.

Preclinical studies in melanoma, glioblastoma, and ovarian cancer have shown that low levels of *p16* and the high expression of cyclin D/Retinoblastoma (Rb) proteins may be thought as biomarkers for the prediction of sensitivity to the CDK4/6i [20,21,22]. In 2017, Wang et al. showed that nearly 85% of breast cancer cells have a normal *Rb* status, ruling out Rb from predictive resistance biomarkers [23]. Aberrations in phosphatidylinositol 3-kinase (PI3K)/protein kinase B (AKT)/mammalian target of rapamycin (mTOR) signalling pathways have been investigated in a metastatic breast cancer setting [24,25,26].

The PALOMA-3 trial assessed endocrine therapy resistance by tumor PIK3CA mutational status in circulating DNA at baseline, but neither the PIK3CA status nor the hormone receptor expression level significantly affected the treatment response [5].

To date, the mechanisms of resistance to CDK4/6i have yet to be clearly identified, and no validated biomarkers have been established for the prediction of the emergence of resistance to CDK4/6i.

To our knowledge, our study is the largest addressing the usefulness and the feasibility of ctDNA analysis in a population of mBC patients to assess KRAS mutations as a biomarker for the prediction of the emergence of resistance to CDK4/6i.

KRAS is the most commonly mutated oncogene in human cancer, with mutations present in approximately 20% of all human cancers, and mutationally activated *RAS*genes have been thought to be the main cause of resistance in several cancers, impacting multiple cellular processes that are critical to tumor progression [27]. Currently, no study, excluding our research, has been focused on breast cancer, and no effective RAS inhibitors have yet been approved [28].

The results obtained by our study support the potential of the significance of basal and sequential KRAS ctDNA assessments for the prediction of therapeutic responses and resistance to CDK4/6i in mBC. A significant difference in PFS was observed in patients with detectable or undetectable ^mut^KRAS in their ctDNA. Changes in KRAS status detected by liquid biopsy provided critical information towards the prediction of therapeutic responses and resistance to treatment: the emergence of ^mut^KRAS ctDNA was significantly associated with resistance to palbociclib, worse PFS, and early recurrence within about 6 months.

Furthermore, liquid biopsy may offer the ability to longitudinally monitor the emergence of resistance mechanisms in real-time and adjust therapy accordingly, allowing us to identify those patients that, within an estimated time of 6 months from the emergence of ^mut^KRAS ctDNA, will most likely have the progressive disease. In the frame of a serial testing for KRAS mutations in liquid biopsies in patients undergoing CDK4/6i, a rise in the circulating mutated ctDNA number of copies/mL might represent a potential marker of cancer cell resistance onset, might anticipate the clinical evidence of disease progression, and thus might allow a rational change in cancer therapy.

## 5. Conclusions

Despite the study’s limitations, such as the retrospective approach and the limited and selected number of enrolled patients, our data confirm that the monitoring of the appearance of tumour molecular alterations (i.e., KRAS) in ctDNA at baseline and during treatment is a promising and reliable tool to detect treatment resistance, anticipating the clinical evidence of disease progression and, thus, allowing us a rational change in cancer therapy.

Further prospective trials are needed to investigate the potential clinical applications of ctDNA analysis in this setting of patients, but our results are in support of the hypothesis that ctDNA^mut^KRAS changes are associated with tumour dynamics, and that they may be used as a biomarker to predict who will take advantage from CDK4/6i, leading to personalized management in HR-positive/HER2-negative mBC patients, decreasing wastes of resources for the National Health System, and ensuring the best quality of life for patients.

## Figures and Tables

**Table 1:** Patients’ baseline clinical characteristics across the cohorts used in the analyses. In the KRAS wild-type cohort, the patients who were initially KRAS wildtype but developed KRAS mutations in the longitudinal sample were included. Abbreviations: ECOG PS, Eastern Cooperative Oncology Group Performance Status; y, years; Nos, not otherwise specified.

Characteristics	Total (*n* = 106)	^mut^KRAS Cohort (*n* = 57)	KRAS-Wild Type Cohort (*n* = 49)	*p*-Value
**Age, median (range), y**	58 (49–74)	56 (49–64)	64 (52–74)	0.140
**Menopausal status, No. (*%*)**				
**Pre-**	31 (*27*)	12 (*21*)	19 (*37*)	
**Post-**	75 (*73*)	39 (*79*)	36 (*63*)	
**ECOG PS, No. (*%*)**				
**0**	87 (*82*)	46 (*81*)	41 (*84*)	0.065
**1–2**	19 (*18*)	5 (*19*)	14 (*16*)	
**Histology, No. (*%*)**				
**Ductal**	93 (*88*)	55 (*96*)	38 (*78*)	0.037
**Lobular**	11 (*10*)	2 (*4*)	9 (*18*)	
**Nos**	2 (*2*)	0	2 (*4*)	
**Metastatic sites, No.**				
**Bone**	100	49	51	0.745
**Liver**	27	22	5	<0.001
**Lymph Nodes**	40	32	8	<0.001
***n* of metastatic sites, No. (*%*)**				
**1**	42 (*40*)	0	42 (*86*)	<0.001
**2**	45 (*42*)	41 (*72*)	4 (*8*)	
**≥3**	19 (*18*)	16 (*28*)	3 (*6*)	

**Table 2:** Univariate and multivariate analysis of the progression-free survival of patients treated with palbociclib and fulvestrant. ECOG PS, Eastern Cooperative Oncology Group Performance Status. LMR, lymphocyte-to-monocyte ratio. HR, hazard ratio. CI, confidence interval.

Parameter		Univariate Analysis			Multivariate Analysis		
		Hazard Ratio	95% CI	*p* Value	Hazard Ratio	95% CI	*p* Value
		All Breast Cancers (*n* = 106)					
Age	>65 years	0.566	0.323–0.989	0.046	1.267	0.684–2.347	0.451
ECOG PS	≥1	0.429	0.195–0.942	0.035			
Histology	Lobular	0.163	0.040–0.669	0.012			
KRAS	Positive	20.746	9.576–44.946	<0.001	3.053	1.112–8.383	0.030
	Copies/ml	1.016	1.012–1.020	<0.001			
Metastases	>1	16.541	7.321–37.372	<0.001			
	Bone	1.120	0.351–3.572	0.848			
	Lymph Nodes	4.334	2.580–7.280	<0.001			
	Liver	2.987	1.792–4.980	<0.001			
Neutropenia	G3	0.803	0.484–1.330	0.393			
	G4	0.399	0.212–0.751	0.004			
	Febrile	0.345	0.125–0.953	0.040			

## Data Availability

All of the source data relating to this manuscript are available upon request.
